# An unexpected finding in a patient with a DDDR pacemaker

**DOI:** 10.1007/s12471-014-0551-y

**Published:** 2014-04-05

**Authors:** A. Bohm, R. G. Kiss, G. Z. Duray

**Affiliations:** Department of Cardiology, Military Hospital, Róbert K körút 44, Budapest, 1134 Hungary

**Fig. 1:**
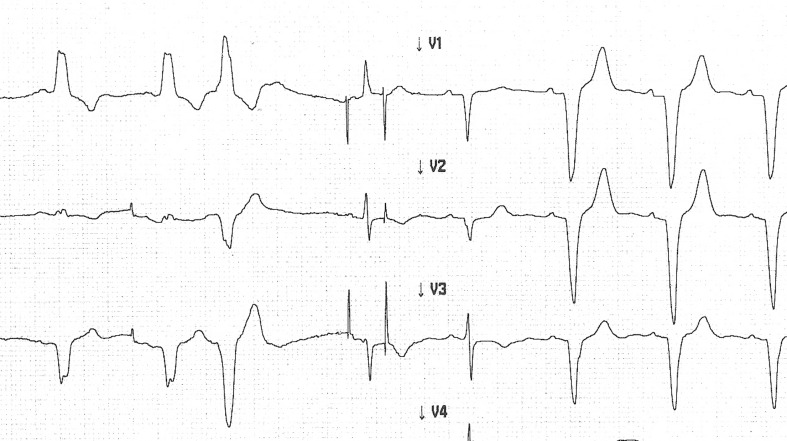
Ventricular sensing failure, when the QRS is narrow

A 77-year-old female patient with sinus bradycardia and left bundle branch block was implanted with a DDD Biotronik Kairos DR pacemaker in 1999, then a replacement for depleted battery was performed in 2007 (Biotronik Axios DR). During our regularly scheduled follow-ups, we found the atrial and ventricular sensing/pacing function to be satisfactory: P: 3.6 mV, R: 9.9 mV, atrial threshold: 0.7 V/0.4 ms, ventricular threshold: 0.5 V/0.4 ms. Parameters were set as follows: basic rate: 60 bpm, hysteresis: −10 bpm, dynamic AV delay: 225 ms, sense compensation: −45 ms, atrial sensitivity: 1 mV, and ventricular sensitivity: 5 mV. During the latest follow-up (14 years after the first implantation), the following recording was made (Fig. 1). What would be your interpretation of this recording?

Answer

You will find the answer elsewhere in this issue.

